# The mediating role of sleep quality and the moderating role of gender and grade in the association between academic stress and psychological health among adolescents in county-level areas of Liaoning Province, China

**DOI:** 10.3389/fpsyg.2026.1705480

**Published:** 2026-02-25

**Authors:** Wenyan Zhang, Rui Wang, Jingmiao Zhang

**Affiliations:** 1 School of Education Science and Technology, Anshan Normal University, Anshan, China; 2 Faculty of Psychology, Beijing Normal University, Beijing, China

**Keywords:** academic stress, adolescents, county-level areas, psychological health, sleep quality

## Abstract

This study examined how academic stress is associated with psychological health among adolescents in county-level areas of Liaoning Province, China, and tested whether sleep quality mediates this association while gender and grade moderate key pathways. A total of 449 students from Grades 7–9 completed validated measures of academic stress, sleep quality (PSQI), and psychological health. Mediation and moderated mediation analyses were conducted using PROCESS (Model 4 and Model 22) with 5,000 bootstrap samples. Academic stress was positively associated with sleep problems (*r* = 0.446, *p* < 0.01) and psychological health problems (*r* = 0.584, *p* < 0.01), while sleep quality showed a strong association with psychological health (*r* = 0.699, *p* < 0.01). Sleep quality partially mediated the association between academic stress and psychological health [*β* = 0.55, *p* < 0.001; 95% CI (0.506, 0.920)]. Grade significantly moderated the stress–health link, with stronger associations observed in Grades 8 and 9 than in Grade 7 (*B* = −0.60, *p* < 0.05). Gender moderated the relationship between sleep quality and psychological health (*B* = −1.59, *p* < 0.05), indicating a stronger association for females.

## 1 Introduction

The rising prevalence of psychological health issues among adolescents has become a pressing global concern. Studies in 2024 indicate that psychological disorders account for at least 45% of the disease burden among individuals aged 10–24, yet only 2% of global health budgets are allocated to psychological health services (Davey, 2020). According to the WHO (2022), one in seven adolescents experiences a psychological health issue, which accounts for 13% of the global burden of disease in this age group. These trends underscore the urgent need to address adolescent psychological health problem on a global scale.

In China, academic competition is intense, which can exacerbate stress among students. According to the data of the Ministry of Education in 2021, the number of students enrolled in middle education is 50.18 million (Mu, 2022), whereas that of general undergraduate students in higher education is 4.45 million (Ministry of Education of the People’s Republic of China, 2021). This means that only a fraction can eventually enter universities, creating fierce pressure on middle school students to excel academically (Li, 2017). As a result, they have to spend more time to complete heavy academic tasks, which puts them under considerable academic stress (Zhao et al., 2015), particularly in less-developed county-level regions where educational resources are scarce, but competition remains high (Zhou et al., 2023; Jiang et al., 2021).

As a result, many Chinese middle school students experience considerable academic stress, generally defined as the psychological strain from excessive academic demands, high performance expectations, and fear of failure (Eduwem and Ezeonwumelu, 2020). Academic stress can manifest in various forms including test anxiety, study overload, and pressure from parents or teachers (Anierobi et al., 2024). Empirical studies have consistently shown that academic stress is associated with a range of negative psychological outcomes such as anxiety and depression (Bhujade, 2017). For instance, a recent study of Chinese adolescents found that academic stress was a significant predictor of depressive symptoms (Jiang et al., 2021). Despite previous studies demonstrating the association between academic stress and psychological health, the mediating mechanism and moderating effect underlying this relationship, especially in the context of China’s competitive educational environment, are less well understood. The present study aims to validate the mediator of sleep quality and moderator of grade and gender between academic stress and psychological health among Chinese adolescents.

### 1.1 Academic stress and psychological health

Academic stress is a form of pressure unique to educational settings, typically arising from various demands of school life, including excessive teacher expectations, intense exam preparation, and heavy homework loads. According to the diathesis-stress model proposed by Schotte and Clum (1987), individuals exposed to prolonged stress without effective coping or problem-solving abilities are at increased risk for developing psychological health disorders. In Western contexts, extensive research has consistently linked high academic stress to adverse psychological outcomes, such as heightened anxiety (Steare et al., 2023), increased depression (Giota and Gustafsson, 2021), suicidal ideation (Gili et al., 2019), reduced happiness (Mahmoodi et al., 2019), and diminished psychological well-being (Aloia and McTigue, 2019). However, findings from Eastern societies have not always aligned with this pattern. In many Asian cultures influenced by Confucian values, education is often viewed as the key to social advancement, and parents typically place a high premium on academic success (Chiu and Wong, 2017). In China, this belief system is particularly prominent, encouraging adolescents to devote considerable time and effort to academic pursuits with the hope of improving their life trajectories. This cultural framework may enhance students’ resilience to academic stress, reducing its psychological impact compared to their Western peers. For example, Jeong (2016) found that academic stress was not a significant predictor of depression among Chinese students. These culturally specific dynamics suggest that the link between academic stress and psychological distress, merits further investigation in the Chinese context.

### 1.2 Sleep quality as mediator of academic stress–psychological health relationship

Sleep quality can be defined as a key determinant of an individual’s overall well-being, including their physical, cognitive, and emotional health. Chronic stress may disrupt sleep, which in turn can impair psychological well-being (Wang and Fan, 2023). According to Lazarus and Folkman's (1984) Stress and Coping Theory, psychological stress arises when individuals appraise a situation—such as academic pressures—as threatening or overwhelming, leading them to feel they do not have sufficient resources to cope with it. This misalignment between academic demands and coping resources can result in poor sleep and then the poor sleep led to psychological and mental exhaustion. In middle school, students facing constant academic demands, such as homework, exams, often perceive these stressors as overwhelming. When they feel they lack the coping mechanisms or resources to manage these pressures, it can lead to poor sleep quality, which then negatively impacts their psychological health (Zhai et al., 2018). Empirical evidence supports this stress–sleep–health pathway: for example, high academic stress has been linked to sleep disturbances among high school students, which in turn contributed to elevated anxiety and depression (Zhang et al., 2022). Moreover, inadequate sleep quality can exacerbate the psychological effects of academic stress, leading to increased emotional instability, irritability, and feelings of helplessness (Zhai et al., 2018). A study by Okun et al. (2018) revealed that adolescents experiencing poor sleep quality due to academic stress were more likely to report symptoms of depression and anxiety. Additionally, research conducted among Chinese adolescents by Li (2017) showed a strong positive correlation between sleep disturbances and depression, with students who experienced poor sleep quality reporting significantly higher levels of psychological distress. Thus, it is plausible that sleep quality mediates the relationship between academic stress and psychological health, such that academic stress leads to poorer sleep, which then elevates the risk of psychological problems.

### 1.3 Grade as moderator of academic stress–psychological health relationship

Another factor that may influence the impact of academic stress is grade level. Middle school students (typically ages 12–15, corresponding to Grades 7–9 in China) are entering adolescence—a developmental period marked by heightened emotional sensitivity and reactivity, which may contribute to imbalanced emotional states (Wang, 2021). During this time, academic pressure is often characterized by negative academic emotions and performance-related stress (Chen et al., 2022), because students’ progress to higher grades, academic workload increases, and the mismatch between their coping capacity and external academic demands becomes more pronounced. According to Developmental Stage Theory (Hayslip et al., 2006), individuals’ responses to stressors vary across developmental stages. It is therefore reasonable to expect that Grade 8 student, who are in the critical transition from Grade 7 to the high-pressure environment of Grade 9, or a Grade 9 student preparing for high school entrance examinations, might experience greater academic stress than a seventh-grade student who is earlier in their educational journey. Prior studies suggest that older middle school students tend to report more academic-related negative emotions. For example, Li et al. (2019) highlighted how academic transitions across developmental stages impact students’ psychological health and performance. For students progressing from Grade 7 to Grade 9, psychological problems (poor psychological health) appears increasingly influenced by academic stress. However, few studies have directly tested whether the association between academic stress and psychological health differs by grade level in early adolescence. This research addresses that gap by examining grade level as a moderator, with the expectation that academic stress may have a stronger detrimental effect on psychological health in higher grades.

### 1.4 Gender as a moderator of sleep quality–psychological health relationship

Gender is an important moderating factor in the relationship between sleep quality and psychological health. Psychological research has consistently documented gender differences in emotional processing, social expectations, and coping mechanisms (Wang et al., 2020). Adolescent girls are generally more prone than boys to sleep-related issues (e.g., insomnia, poor sleep satisfaction) and internalizing symptoms such as anxiety and depression (Rajab et al., 2021; Lindberg et al., 1997; Conklin et al., 2018). Notably, girls appear to be more psychologically sensitive to the effects of poor sleep, meaning that sleep disruptions have a stronger negative impact on their psychological well-being. This heightened vulnerability stems from both biological and psychosocial factors. For instance, girls undergo unique pubertal changes, including menstruation and hormonal fluctuations—particularly in estrogen and progesterone—that can disrupt sleep duration and quality (Urrila et al., 2015; Sharkey et al., 2014; Zheng et al., 2015). These disturbances may activate the hypothalamic–pituitary–adrenal (HPA) stress axis, to which girls are especially reactive (Handa et al., 1994). Moreover, girls are more emotionally responsive to academic and social stressors, reporting higher levels of anxiety and psychosomatic symptoms in school settings (Giota et al., 2017). Lifestyle differences, such as physical activity, diet, and screen time (e.g., social media, gaming, watching videos), may further contribute to disparities in sleep and psychological health outcomes between genders (Cairns et al., 2014). Therefore, understanding how gender moderates the impact of sleep quality on psychological health is critical, as it highlights the need for gender-sensitive psychological health interventions. This is especially important in county-level regions, where traditional gender roles and expectations may further shape girls’ psychological responses to sleep disturbances.

### 1.5 Conceptual framework

**Figure 1:**
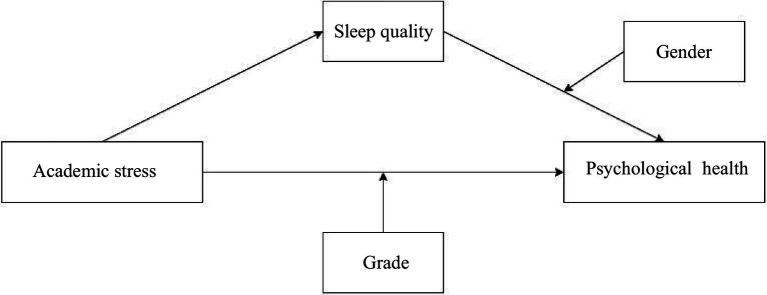
Conceptual framework.

According to the above analyzing, the following conceptual framework is proposed: Figure 1. In this model, academic stress is expected to have both a direct effect on adolescents’ psychological health and an indirect effect through sleep quality (mediating pathway). Moreover, this research posits that the strength of these pathways is contingent on the student’s gender and grade level. In summary, this study aims to test a moderated mediation model in which sleep quality mediates the effect of academic stress on psychological health, with grade level and gender acting as moderators of the stress–health and sleep–health relationships, respectively. The conceptual framework of this research is showed in Figure 1.

## 2 Methods

A convenience cluster sampling strategy was adopted in this study due to the need for institutional access to middle schools. Schools were approached through county-level educational administrative coordination and existing school cooperation channels to facilitate questionnaire distribution. This study primarily focused on distributing questionnaires in middle schools located in the northern, central, and southern county-level areas of Liaoning Province, China. Prior to distribution, the study received ethical approval from the Academic Committee of Anshan Normal University.

### 2.1 Participants

Table 1 presents the demographic analyze of the sample by region, grade, and gender. In brief, of the 449 respondents, 32% were in 7th grade (typically ages 12–13), 28% were in 8th grade (ages 13–14), and 40% were in 9th grade (ages 14–15). The northern Liaoning school contributed the largest number of participants (*n* = 294), while the central and southern region schools contributed 98 and 57 students, respectively. The gender ratio was roughly similar across regions and grades. This sampling strategy yielded a broad cross-section of adolescents in county-level areas of Liaoning.

**Table 1:** Descriptive analyze.

Region	Male	Female	Total	%	Grade 7	Grade 8	Grade 9
North Liaoning	165	129	294	65.50%	125	111	58
Central Liaoning	49	49	98	21.80%	15	6	77
South Liaoning	29	28	57	12.70%	4	7	46
Total	243	206	449	100%	144	124	181

### 2.2 Measures

#### 2.2.1 Pittsburgh Sleep Quality Index

Sleep quality was measured with Pittsburgh Sleep Quality Index. A widely used questionnaire, the (PSQI) comprises 19 items with different response formats (5-point Likert scales and open-ended questions) concerning perceived sleep quality, habitual sleep schedules, and common sleep problems. The instrument measures seven dimensions (subjective sleep quality, use of sleep medications, and daytime dysfunction) with scores between 0 and 3 obtained from specific items for each dimension. These component scores are summed to produce a global sleep quality score ranging from 0 to 21, where higher scores indicate worse sleep quality (i.e., more severe sleep problems). A cut-off of 5 demonstrated good sensitivity and specificity in discriminating between poor sleepers and good sleepers, and the Italian version of the PSQI showed good reliability (*α* = 0.83) and was able to distinguish patients with sleep disorders and healthy control. In this study, sleep quality was assessed using the Chinese version of the Pittsburgh Sleep Quality Index (PSQI), which has been validated in Chinese populations. In accordance with the standard scoring protocol for this scale, the global PSQI score was used for analysis (Buysse et al., 1989; Larche et al., 2021; Huang et al., 2021). In this study, the scale reliability was acceptable with 0.899.

#### 2.2.2 Academic stress

Academic stress was measured using the Academic Stress Questionnaire for Middle School Students [developed by Xu et al. (2010)]. This scale consists of 21 items covering four domains of academic pressure: parental pressure, teacher pressure, peer/social pressure, and self-imposed pressure. Students rated each item on a 5-point Likert scale from 1 (“strongly disagree”) to 5 (“strongly agree”), with higher scores reflecting greater perceived academic stress (Xu et al., 2010; Shih Ching et al., 2024). In this study, the total score of the Academic Stress Questionnaire was computed for subsequent analysis, and the academic stress scale demonstrated good reliability, with a Cronbach’s alpha of 0.902.

#### 2.2.3 Psychological health

This study adopted the Mental Health Rating Scale for Middle School Students. The Mental Health Rating Scale for Middle School Students, developed by Wang et al. (1998), is a widely used tool in China for measuring mental health among adolescents. In this study, we use this scale to assess psychological health, as it comprehensively captures key aspects of emotional and psychological well-being, including symptoms of anxiety, depression, and general emotional distress, which are central to understanding an individual’s psychological health with higher values corresponding to worse psychological health problems. The scale consists of 60 items and is designed to comprehensively assess the psychological problems. Each item is rated on a 5-point Likert scale, with five levels of severity: 1 = “not at all,” 2 = “a little,” 3 = “moderate,” 4 = “severe,” 5 = “extremely severe” (Wang et al., 1998; Chen, 2023). In the present study, the mean total score of the Mental Health Rating Scale for Middle School Students was calculated for analysis, and the internal consistency of the scale was acceptable, with a Cronbach’s alpha coefficient of 0.983, indicating high reliability.

### 2.3 Data analysis

Preliminary analyses, including descriptive and correlational, of the study variables (academic stress, sleep quality, psychological health, gender, and grade) are carried out using SPSS 24.0. Mediation and moderation analyses are performed using the SPSS macro-Process. First, the mediation model is examined to identify the mechanism through which sleep quality mediates the relationship between academic stress and psychological health. The mediating effect is determined by using the macro- Process (Model 4), the analysis employed 5,000 bootstrap samples to test the indirect effect. Second, the moderated mediation effects are tested by taking micro-process (Model 22), if the coefficient for the interaction between academic stress and grade as well as between sleep quality and gender, is significant, then the moderation effect can be identified. Specifically, the moderating effect of grade on the relationship between academic stress and psychological health is examined, while the moderating effect of gender on the relationship between sleep quality and psychological health is tested.

## 3 Findings

### 3.1 Preliminary analyses

The potential for common method bias was tested using principal component analysis. Sixteen factors had eigenvalues above 1, with the first factor explaining 40.24% of variance, below the 50% threshold (Shrestha, 2021), indicating no serious bias. Descriptive statistics (Table 2) showed moderate academic stress (M = 46.38, SD = 13.01), average PSQI score of 3.69 (SD = 3.18), and mean psychological health score of 85.00 (SD = 37.85), suggesting moderate level. Since the normality tests indicated that the data were non-normally distributed (*p* < 0.05), Spearman’s rank correlation was employed to calculate the correlation coefficients between variables. Correlations supported hypotheses: academic stress was positively associated with sleep problems (*r* = 0.42, *p* < 0.001) and psychological issues (*r* = 0.51, *p* < 0.001), while sleep quality strongly correlated with psychological health (*r* = 0.68, *p* < 0.001).

**Table 2:** Descriptive statistics and correlation matrix of variables (*n* = 449).

Variable	M	SD	1	2	3	4	5
1. Academic stress	46.38	13.01					
2. Sleep quality	3.69	3.18	0.42**				
3. Psychological health	85.00	37.85	0.51**	0.68**			
4. Grade-1	0.32	0.47	−0.11*	−0.07	−0.05		
5. Grade-2	0.28	0.45	−0.03	−0.02	0.12*	−0.42**	
6. Gender	0.54	0.50	−0.06	−0.07	−0.14**	0.001	0.01

M is mean; SD is Standard Deviation; Grade-1 is a dummy variable: Grade 7 = 1, Grade 8 and Grade 9 = 0; Grade-2 is a dummy variable: Grade 8 = 1, Grade 7 and Grade 9 = 0; Gender is a dummy variable: Male = 1, Female = 0; *p < 0.05, **p < 0.01.

### 3.2 Sleep quality as mediator

As the research variables exhibited a non-normal distribution, the PROCESS macro developed by Hayes (2018) was employed to conduct the moderation analysis. According to Hayes (2018), the bootstrapping method utilized by PROCESS is a non-parametric resampling technique that does not require the data to follow a strict normal distribution (Preacher and Hayes, 2008). In this study, the number of bootstrap samples was set to 5,000, and the significance of the effects was determined by observing whether the 95% confidence intervals included zero. This approach demonstrates high statistical robustness when handling non-normal data (Hayes and Rockwood, 2017).

To examine whether sleep quality mediates the relationship between academic stress and psychological health, a mediation analysis was conducted. The results showed that academic stress significantly predicted sleep quality (*B* = 0.11, *t* = 10.53, *p* < 0.001) in the first regression model, the positive coefficient indicates that higher academic stress was associated with higher PSQI scores—that is, greater academic stress led to poorer sleep quality (more sleep disturbances). In the second model, academic stress also showed a significant direct effect on psychological health (*B* = 0.99, *t* = 9.91, *p* < 0.001) when not accounting for sleep, meaning that students under higher academic stress tended to have more psychological health problems. In the full mediation model (third regression), sleep quality remained a strong independent predictor of psychological health (*B* = 6.53, *t* = 15.99, *p* < 0.001). Importantly, even after including sleep quality in the model, academic stress still had a significant (though reduced) effect on psychological health (the direct effect). In addition, the analyzing form bootstrapped 95% confirms the significant indirect effects of sleep quality in the relationship between academic stress and psychological health [95% CI (0.506, 0.920)]. These results indicate that sleep quality partially mediates the relationship between academic stress and psychological health (see Table 3).

**Table 3:** Results of moderation analysis.

Dependent variable	Predictor variable	*R*	*R*^2^	*F*	*B*	*t*
Sleep quality	Academic stress	0.45	0.20	22.48**	0.10	6.20**
	Grade-1				−0.28	−0.87
	Grade-2				−0.22	−0.66
	Academic stress * Grade-1				0.01	0.36
	Academic stress * Grade-2				0.03	1.10
Psychological health	Academic stress	0.78	0.60	82.81**	1.20	8.7**
	Sleep quality				6.52	16.2**
	Grade-1				3.39	1.24
	Grade-2				8.41	2.98*
	Academic stress * Grade-1				−0.60	−2.7*
	Academic stress * Grade-2				−0.20	−0.98
	Gender				−2.59	−1.13
	Sleep quality * Gender				−1.59	−2.20*

Grade-1 is a dummy variable: Grade 7 = 1, Grade 8 and Grade 9 = 0; Grade-2 is a dummy variable: Grade 8 = 1, Grade 7 and Grade 9 = 0; Gender is a dummy variable: Male = 1, Female = 0; *p < 0.05, **p < 0.01. *β* is Standardized, *B* is unstandardized coefficients.

### 3.3 Moderated mediation analysis: the role of grade and gender as moderators

#### 3.3.1 Moderating role of grade

**Figure 2:**
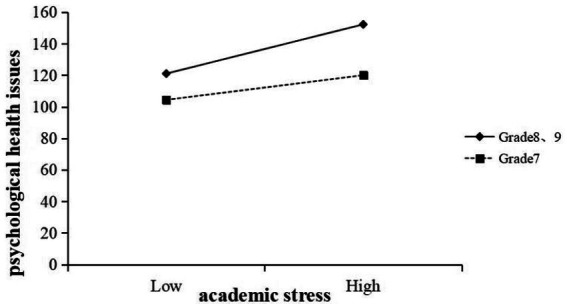
Grade moderates the relationship between academic stress and psychological health issues.

To test the moderating role of grade between academic stress and psychological health, this research analyzes the interaction terms between academic stress and the grade dummy variables in regression model. The analysis revealed a significant interaction effect for Academic Stress × Grade-1 (*B* = −0.60, *t* = −2.70, *p* < 0.05) (in Table 4), indicating that the relationship between academic stress and psychological health was significantly weaker for Grade 7 students compared to the reference group (Grade 8, 9). In contrast, the interaction between academic stress and Grade-2 was not significant (*B* = −0.2, *t* = −0.98, *p* > 0.05), suggesting no meaningful difference between Grade 8 and Grade 7, 9 in this association. This research probed the interaction by examining the simple slopes of academic stress on psychological health for each grade. These results are depicted in Figure 2. For Grade 8, 9 students (the reference group), academic stress had a positive association with psychological health problems (simple slope *β* = 1.20, *p* < 0.001), meaning higher stress corresponded to more symptoms—this is consistent with the overall main effect. In contrast, for Grade 7 students, the slope was about half as steep (simple slope *β* = 0.60, *p* < 0.05). This indicates that for eighth and ninth graders, increases in academic stress were associated with a much larger increase in psychological health issues compared to seventh grades. In practical terms, a unit increase in academic stress score had a smaller detrimental impact on psychological health in Grade 7 than in Grade 8 or 9. Grade 8, 9 students thus appear particularly vulnerable to the effects of academic stress. Statistically, the difference in slopes between Grade 7 and Grade 9 was significant (as indicated by the significant interaction term), whereas the slope difference between Grade 8 and Grade 9 was not. This suggests a moderating effect of grade level: the strength of the association between academic stress and psychological health problems was greater for students in Grades 8 and 9 than for those in Grade 7. As illustrated in Figure 2, psychological health issues increased more steeply with academic stress among Grade 8 and 9 students, whereas the increase was more modest for Grade 7 students. These findings indicate that younger adolescents may be less vulnerable to the psychological impacts of academic stress than their older peers.

**Table 4:** Mediation analysis.

Outcome variable	Predictor variable	*R*	*R*^2^	*F*	*B*	*t*
Sleep quality	Academic stress	0.45	0.20	110.92**	0.11	10.53**
Psychological health	Academic stress	0.76	0.58	309.04**	0.99	9.91**
	Sleep quality				6.53	15.99**

**p < 0.01.

#### 3.3.2 Moderating role of gender

**Figure 3:**
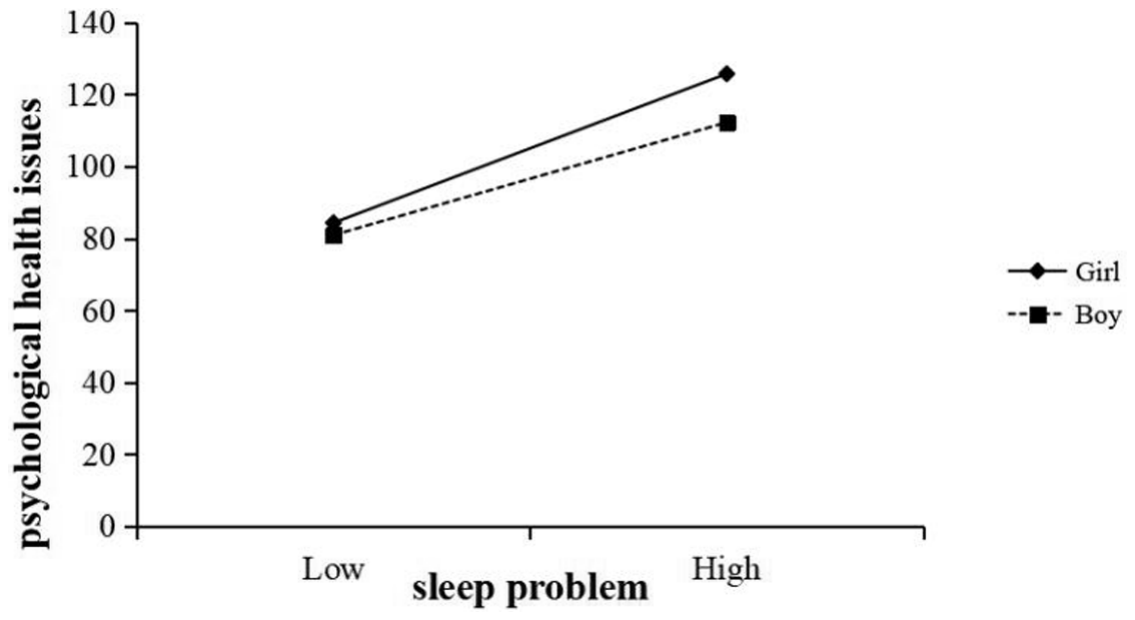
Gender moderates the relationship between sleep problem and psychological health issues.

This research also examined whether gender moderates the relationship between sleep quality and psychological health. The interaction term Sleep Problem × Gender was statistically significant (*B* = −1.59, *t* = −2.20, *p* < 0.05) (in Table 4), indicating that the strength of this relationship differed by gender. Specifically, the positive coefficient suggests that the impact of poor sleep on psychological health was greater for females (coded as 0) than for males (coded as 1). To interpret this interaction, this study conducted a simple slopes analysis for males and females separately. Figure 3 illustrates these relationships. For female students, poorer sleep quality was associated with a pronounced increase in psychological health problems (simple slope *β* = 6.52, *p* < 0.001). For male students, poor sleep was also linked to more psychological issues, but the slope was more modest (*β* = 4.93, *p* < 0.001). Both slopes were significantly positive, reflecting that in all gender, getting poorer sleep corresponded to worse psychological health. However, the slope for females was significantly steeper than that for males, consistent with the significant interaction term. This means that the impact of sleep quality on psychological well-being was greater for girls than for boys.

In summary, our moderated mediation analysis confirmed that sleep quality serves as a partial mediator between academic stress and psychological health, and that this mediation process is contingent on both grade level and gender. Academic stress had the strongest adverse effect on psychological health among Grade 9 students, with a similarly high impact observed in Grade 8, while the association was weaker in Grade 7. Additionally, poor sleep quality exerted the most deleterious effect on psychological health among female students. The overall model accounted for a substantial portion of the variance in psychological health (adjusted *R*^2^ = 0.60 in the final moderated mediation model shown in Table 4), indicating that academic stress, sleep quality, and their interactions with demographic factors are important contributors to adolescents’ mental health in this context.

## 4 Discussion

This section discussed each of the major findings in turn, in light of existing theories and research.

### 4.1 Academic stress and psychological health

The findings of this study demonstrate that academic stress has a significant negative impact on adolescents’ psychological health, which is consistent with previous research emphasizing the detrimental effects of academic pressure on mental well-being, including symptoms of depression and anxiety (Jiang et al., 2021). This result aligns with the transactional model of stress and coping (Lazarus and Folkman, 1984), which suggests that stressors such as academic overload can trigger emotional strain and psychological maladjustment. Furthermore, the result supports the diathesis-stress framework (Schotte and Clum, 1987), indicating that students who lack effective coping mechanisms are particularly vulnerable under prolonged academic stress. Although previous studies in Eastern societies, such as the work of Jeong (2016) and Chyu and Chen (2022), have suggested that cultural values might buffer the psychological impact of academic stress, the current findings suggest that this protective effect may be weakening in contemporary Chinese adolescents. This could be due to increasing academic competition, reduced leisure time, or changing societal expectations. Hence, while cultural factors remain important, the present study underscores the urgent need to address academic stress as a significant predictor of adolescent psychological problems, particularly in rapidly changing educational environments like those in China.

### 4.2 Sleep quality as a mediator

The study confirmed that sleep quality significantly mediates the effect of academic stress on adolescents’ psychological health. Academic stress impaired sleep, which in turn heightened depression and anxiety symptoms, aligning with prior findings (Zhang et al., 2022; Okun et al., 2018; Li, 2017; Zhai et al., 2018). These results reinforce the role of poor sleep as a key pathway linking academic burden and mental distress. Theoretically, this mechanism is explained by Lazarus and Folkman’s (1984) Stress and Coping Theory: when students perceive academic demands as exceeding coping capacity, sleep disruption occurs, which undermines emotional regulation and cognitive functioning. This cycle increases vulnerability to psychological problems, illustrating how poor sleep acts both as a consequence of academic stress and a contributor to deteriorating mental health.

### 4.3 Gender as a moderator

The present study found that gender significantly moderates the relationship between sleep quality and psychological health. Specifically, the negative impact of poor sleep on psychological well-being was found to be stronger among female adolescents than male peers. This result is consistent with previous research showing that girls are more likely to report sleep disturbances, including insomnia and sleep dissatisfaction, as well as internalizing symptoms such as anxiety and depression (Rajab et al., 2021; Lindberg et al., 1997; Conklin et al., 2018). This heightened sensitivity to poor sleep among girls may be explained by both biological and psychosocial factors. Biologically, girls experience unique pubertal transitions and more pronounced hormonal fluctuations (e.g., estrogen and progesterone), which can disrupt sleep and increase emotional reactivity (Sharkey et al., 2014; Urrila et al., 2015). Psychosocially, adolescent girls are often more emotionally responsive to academic and social stressors and may be more attuned to changes in their physical and emotional states (Giota et al., 2017). Moreover, lifestyle factors such as differences in screen time, physical activity, and dietary patterns may further amplify the gender gap in sleep and psychological health outcomes (Cairns et al., 2014). These findings underscore the importance of developing gender-sensitive psychological health strategies that consider how girls may be particularly vulnerable to the psychological consequences of inadequate sleep, especially in county-level areas where traditional gender norms may reinforce these sensitivities.

### 4.4 Grade as a moderator

The study found that grade level significantly moderates the link between academic stress and psychological health. Grade 7 students showed a weaker association, while Grades 8 and 9 demonstrated stronger psychological sensitivity to stress. This pattern aligns with prior studies noting developmental stages shape stress responses (Li et al., 2019; Chen et al., 2022; Wang, 2021). Older middle school students face heightened academic demands, exam pressure, and future placement concerns, which amplify psychological strain. According to Developmental Stage Theory (Hayslip et al., 2006), stress responses vary with maturity; students in transitional grades often lack sufficient emotional regulation, making them more vulnerable. Grade 8 and 9 students may thus experience a “double burden” of increasing academic expectations and ongoing emotional adjustment, whereas Grade 7 students encounter comparatively fewer pressures and exhibit greater resilience. These findings highlight the need for grade-specific interventions. Schools and practitioners should prioritize coping strategies and emotional support in later middle school years, recognizing Grades 8 and 9 as critical periods for academic stress–related psychological challenges.

## 5 Conclusion

This study provides empirical support for a moderated mediation model linking academic stress, sleep quality, and psychological health in Chinese adolescents. By demonstrating that sleep quality mediates the effect of academic stress on psychological health—and that this mediation is contingent on both grade and gender—it deepens our understanding of the contextual factors that influence adolescent well-being. Specifically, students in higher grades and female students appear more susceptible to the psychological toll of academic pressure and poor sleep. These findings call for proactive, tailored interventions that integrate academic stress management with sleep-focused psychological health strategies.

## 6 Limitation

This study employed a cross-sectional design, which precludes causal inferences. The observed associations between academic stress, sleep quality, and psychological health are correlational, and longitudinal studies are needed to establish causal relationships. Additionally, the sample was drawn from county-level areas in Liaoning Province, which represent less developed cities. Future studies could include samples from more developed cities to enable comparative research between developed and less developed areas.

## Data Availability

The raw data supporting the conclusions of this article will be made available by the authors, without undue reservation.
